# The Drink Question in Switzerland

**Published:** 1893-05-06

**Authors:** 


### The Drink Question in Switzerland.

Switzerland escapes many of the troubles which beset larger States. Supported by various great powers as a bulwark against each other, it has no foreign policy to speak of. Poor and provident, with few great industries and fewer temptations to speculation, it hardly understands the meaning of financial crisis. Labour troubles affect it little, and though it abounds with Socialists, Anarchists, and Nihilists, few of these are of native growth. Behind the shelter of its impregnable mountains, and its still more impregnable neutrality, "men are bold and freely say their say," especially if they are men of alien birth, saying their say about their own nationality, and not about that whose hospitality they enjoy. To us, surrounded with great problems upon every hand, it might seem that the Federated Cantons had little need for a national assembly, and that all their affairs might be regarded as merely parochial. Yet, as a nation, Switzerland has been confronted with one of the problems which most nearly affect ourselves—the drink question; and it may be worth while, in face of the agitation for and against the local veto on liquor-selling, to see what has been done by the little Helvetian Republic.

It is only a few years since the Swiss felt themselves compelled to face a difficulty which we thought we had settled long ago, with the result of creating a privilege in what we meant as a restriction. Till lately small distilleries existed everywhere. The peasant had his still, where he made coarse "schnaps" too crude for sale to the taverns, and therefore the more readily consumed at home. The picture drawn in 1864 by an observer, Dr. Schild, of the moral results of the existence of these small stills is sufficiently alarming. "In the fields, in the houses, and in the workshops we are forced to the conclusion that 'schnaps' is the daily drink morning and evening. The many stills bring 'schnaps' in plenty into the peasants' houses, and the workmen must content themselves with it, and *nolens volens*, accustom themselves to it. Even children are often given a little drop, and this becomes a large drop, and then a little glass, and finally a whole glass. No one should be surprised that drinking has become so common in peasants' houses; that, contrary to former customs and habits, peasants' sons appear in taverns after having become at home regular drinkers. Many illustrations are familiar here in the country of respectable peasant families who have become distillers, and who have been ruined by the immoral consequences of 'schnaps' drinking, despite the material advantages which they have gained." In short, national drunkenness was becoming the curse of the country.

The extent of this private distillation may be inferred from the fact that since 1887, when the new laws came in force, fourteen hundred distilleries, large and small, have been bought out, and between sixty and seventy remain. The latter, however, can sell their products only to the monopoly administration. This has been the chief remedy essayed—to make the State the sole vendor of alcohol. In Britain, as we know, while such a proposal would be welcomed by many as tending to prevent the consumption of that raw, adulterated liquor, little short of poison, which is sold (at an enormous profit) by unscrupulous publicans to our working people; it would, on the other hand, be decried by those to whom alcohol in any form is anathema, as making the State the direct patron and encourager of drunkenness. But in Switzerland there is practically no teetotal party. There does, indeed, exist a temperance society, the "Blue Cross," but its numbers are small (5,348 in 1890), and its influence on practical politics infinitesimal. The Swiss Government has proceeded on the principle that it is with alcoholism, the disease arising from immoderate indulgence in intoxicants, and its evil effects on society that they have to deal, not with the consumption of fermented liquors in the abstract. A proposal, therefore, to limit drinking by limiting the number of taverns was defeated, partly because it was thought that the suggestion tended to interference with that freedom of trade guaranteed by the Federal Constitution of 1874, and partly, also, because it was felt that this proposal did not touch the root of the evil. We in England know that it does not, and that its adoption by our own nation has led to the creation of a powerful and privileged class of publicans, who regard their licence, not as a restriction, but an investment, which, if taken from them, must be redeemed at considerable cost. The Swiss monopoly administration has avoided that blunder. The administration is itself the wine and spirit merchant of the Republic, and no private individual has a right or profit in the trade.

This, however, is not all. A secondary, yet most important, step has been taken, which, unfortunately, public opinion renders almost impossible here. The Government has tried to encourage the drinking of beer and wine as a preventive to the continual consumption of "schnaps." Wine and beer are, of course, both of the lightest kind, the "ordinaire" and "bock" of France, not the heady and stupefying liquors to which we extend the title. Now, while British Governments have given or withheld favourable tariffs to suit political convenience or gratify patriotic prejudice, they have never, so far as we know, encouraged the consumption of certain liquors for the sake of national health. In fact, they dare not do it. If they proposed to make it possible for an Englishman to drink light, sound wine as cheaply as a Frenchman all parties would condemn them—the abstainers for encouraging drinking, the great brewers and distillers for favouring foreign products to the injury of native interests, the publicans for trying to corrupt the Englishman's native preference for beer, the Scotchman's for peat-flavoured whisky, and the Irishman's for smoky potheen, while the politicians and the ambassadors would demand that equal privileges should be extended to every wine-producing country under the sun. And then the Chancellor of the Exchequer would ask where his revenue was to come from. Certainly those who try to deal with national sobriety in this country have a hard task. The difficulties of the old man with the ass were as nothing to it. They are taunted with the National Drink Bill, yet if they essayed to lessen it by reducing duties, those who taunt them would be the first to complain; while if they try to encourage the drinking of wine in order to prevent the drinking of spirits, they are simply accused of favouring the rich and bearing hardly on the poor.

But there is practically no objection to the State being the national vendor of intoxicants. It could then regulate the quality as well as the price. The profits which now go into the publican's pocket would become a portion of the national revenue, and thus compensate for any loss attributable to decrease of consumption, which would otherwise have to be made up by taxes on some other article. Great as is the praise bestowed on the Gothenburg system, it has been found in Gothenburg that the municipality is too small a body to act with entire devotion to the public good. There are local interests to serve, and well-meaning councillors did not look too hardly on a large drink bill, which made possible some great city improvement. Under this leniency the clause which ordered that eating and drinking should go together was largely ignored; at least, the proportion was that of Falstaff's friends—"a poor pennyworth of bread to this intolerable deal of sack." The Swiss Government avoided this danger by devoting one-tenth of the profits of liquorselling to the aid of societies which are trying to suppress drunkenness, and to institutions for the care of the sick and the insane who are often its victims. This sum amounts to between 600,000 and 700,000 francs annually. In pursuance also of its desire to introduce wholesome and comparatively non-intoxicating liquors for the crude and dangerous "schnaps," a considerable sum has been spent on vine culture and experiments in the manufacture of good light wines. At the same time the taxes on wine and beer have been lowered while the duty on distilled liquor has been nearly trebled.

The result has been a great decrease in the consumption of the latter. Before the monopoly administration started it was increasing rapidly. The figures given by Herr Milliet, of Berne, are these: 1882, 9·4 litres per head; 1885, 10·26. Since the monopoly there has been a great and increasing diminution: 1890, 6·27; 1891, 6·32. Against this must be set a slight increase in the use of wine and a considerable increase in that of beer, and we must also allow for the fact that before the monopoly some proportion of the liquor distilled in Switzerland, and here credited to the consumption of the native population, was actually smuggled into foreign countries. Under the present system smuggling is more carefully watched than was possible in the old days of private stills, and it has sunk to insignificant proportions; we may assume that the latest statistics tell of an amount which is practically all drunk in the country. Still, competent observers reckon that the decrease in the use of spirituous liquors amounts to 25 per cent.

To comment on the desirability of such a result is needless. It has been brought about in a very short time, and apparently without creating any public excitement. It has been due to a large extent to the pursuance of a policy of moderation, which has kept a mean between the assumption that alcohol is an unmitigated curse and the other position that the rights of the publican are sacred and unassailable. The private spirit-seller has indeed been eliminated, but in no spirit of malignity, and the action of the State has been directed not against the café and the tavern, where men assemble for society and amusement, but against alcoholism as a pernicious disease to be dealt with as we deal with small-pox or cholera. The mere reduction of the number of taverns was found to be a futile measure; a larger business was done at those which were allowed to remain. But increased price of the spirit sold, a high standard of quality in it, the destruction of private profit from the sale, and the increased facilities for obtaining pure liquors of a lighter and less injurious nature, these have largely settled the drink question in Switzerland. Would they not do much to solve the problem as it faces us in Britain to-day?

